# HIV, Inflammation, and Immunometabolism: A Model of the Inflammatory Theory of Disease

**DOI:** 10.3390/v17060839

**Published:** 2025-06-11

**Authors:** Eman Teer, Nyasha C. Mukonowenzou, M. Faadiel Essop

**Affiliations:** 1 Centre for Cardio-Metabolic Research in Africa, Department of Physiological Sciences, Faculty of Science, Stellenbosch University, Stellenbosch 7600, South Africa; eman.ab.teer@gmail.com (E.T.); 25707264@sun.ac.za (N.C.M.); 2 Centre for Cardio-Metabolic Research in Africa, Division of Medical Physiology, Faculty of Medicine and Health Sciences, Stellenbosch University, Cape Town 8000, South Africa

**Keywords:** HIV infection, immune activation, chronic inflammation, molecular mechanisms, immunometabolism, inflammatory theory of disease

## Abstract

Inflammation is a crucial component of the immune response essential for host defense and tissue repair. However, when the immune response becomes dysregulated, it can contribute to the pathogenesis of chronic diseases. While acute inflammation is a short-lived, protective response, chronic inflammation is sustained over time and can lead to immune dysfunction, tissue damage, and disease progression. The chronic inflammation theory of disease suggests that persistent immune activation/inflammation underlies both infectious and non-infectious conditions and serves as a unifying mechanism across distinct pathological states. In this review article, we argue that human immunodeficiency virus (HIV) infection represents a prime model for studying chronic inflammation, and that despite effective viral suppression with antiretroviral therapy (ART), people living with HIV (PLWH) exhibit persistent immune activation, systemic inflammation, and an increased risk of cardiovascular, metabolic, and neurodegenerative diseases. Here, the interplay between microbial translocation, immune dysregulation, and metabolic reprogramming fuels a state of chronic inflammation that accelerates disease progression beyond HIV itself. Key factors such as T-cell exhaustion, persistent monocyte/macrophage activation, and immunometabolic dysfunction contribute to such a sustained inflammatory state. This review explores the molecular and cellular mechanisms driving chronic inflammation in HIV infection with a focus on immunometabolism and its implications for broader inflammatory diseases. By understanding such pathways, we can identify novel therapeutic targets to mitigate inflammation-driven disease progression not only in HIV but across a spectrum of chronic inflammatory conditions.

## 1. Introduction

Inflammation is a fundamental immune response that acts as a double-edged sword, i.e., it is essential for host defenses, tissue repair, and immune surveillance, though potentially harmful if dysregulated [1]. The distinction between acute and chronic inflammation lies in their duration, immune cell involvement, and ultimate consequences [2]. Here, acute inflammation is a relatively short-lived, rapid response to infections, injuries, or toxins, aimed at eliminating pathogens, clearing damaged cells, and initiating tissue repair [2,3]. In contrast, chronic inflammation is a prolonged response that arises when the immune system fails to resolve an initial insult and/or remains persistently activated due to infections, autoimmunity, or metabolic dysfunction [1,4]. Moreover, chronic inflammation—unlike its acute counterpart—can lead to tissue damage, fibrosis, and disease progression [5,6].

Inflammation is traditionally recognized as a hallmark of infectious diseases. However, advances in molecular and epidemiological research suggest that chronic, low-grade inflammation is a central factor in a broad range of non-infectious ailments such as cardiovascular diseases (CVDs), cancer, diabetes, and neurodegenerative disorders, thus potentially serving as a unifying mechanism across apparently distinct pathological conditions [1,4]. This concept is often referred to as the chronic inflammation theory of disease and postulates that persistent inflammation acts as a key driver of disease initiation and progression, making it a crucial target for understanding and managing chronic illnesses [7].

One of the most well-studied examples of chronic inflammation as a disease driver is human immunodeficiency virus (HIV) infection [8], where persistent immune activation and systemic inflammation contribute to disease progression and non-AIDS (acquired immunodeficiency syndrome)-related comorbidities [9]. Here, individuals with HIV often exhibit elevated levels of inflammatory markers, immune dysfunction, increased CVD risk, neurocognitive impairment, and metabolic disorders despite effective antiretroviral therapy (ART) suppressing viral replication [9,10]. Such chronic immune activation is driven by ongoing viral replication in reservoirs, microbial translocation, and immune dysregulation and renders HIV a compelling model for understanding the long-term consequences of inflammation in human disease [9,11,12].

The pathophysiology of chronic inflammation is complex, involving intricate interactions between the host immune system and various inflammatory triggers [1]. A crucial aspect of this process is the interplay between the innate immune system’s early response and the subsequent activation of adaptive immunity [5]. Understanding these mechanisms is therefore essential for developing therapeutic strategies to mitigate the detrimental effects of chronic inflammation and thereby lower the burden of inflammatory-driven diseases.

**Figure 1:**
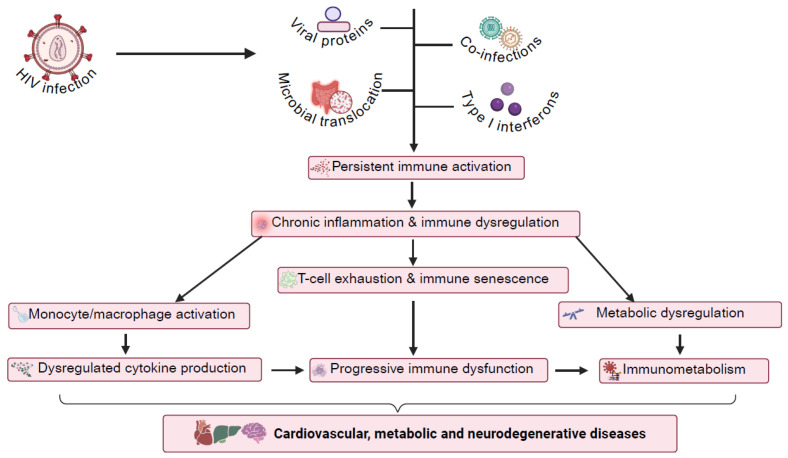
Links between HIV infection and immune dysregulation and chronic inflammation. The consequences of T-cell exhaustion, persistent monocyte/macrophage activation, immune cell senescence, and immunometabolic dysfunction in the development of non-AIDS comorbidities are indicated. Abbreviations—HIV: human immunodeficiency virus. Image created using BioRender.

The inflammatory theory of disease indicates that chronic, unresolved inflammation is a major driver of disease progression in various pathological complications [1,7]. HIV is a compelling model that supports this theory as immune activation plays a pivotal role in terms of disease outcomes [12]. Studies in non-human primates infected with the simian immunodeficiency virus provide a unique comparative framework for HIV pathogenicity. Here, for non-pathogenic hosts such as the African green monkey, immune activation remains controlled, CD4 T-cell restoration occurs, and disease progression is absent despite stable viral replication [13,14]. In contrast, pathogenic species such as the Rhesus macaque exhibit persistent immune activation, lack CD4 T-cell recovery, and experience immune dysfunction, thereby leading to the progression to AIDS [15]. These differences highlight the crucial role of the early innate immune response in determining the severity of disease onset and progression. For example, non-pathogenic hosts establish a relatively early anti-inflammatory milieu that is characterized by increased IL (interleukin)-10 production, decreased T-cell activation, and resistance to microbial translocation [16,17]. In contrast, persistent immune activation, increased apoptosis, and microbial translocation can fuel a cycle of chronic inflammation in pathogenic hosts, contributing to systemic immune dysfunction and end-organ disease [18,19]. This comparative model underscores how immune activation and chronic inflammation are central to HIV pathogenesis, reinforcing the broader inflammatory theory that chronic immune dysregulation underlies several disease states, including CVD, neurodegeneration, and metabolic disorders [9,11] (Figure 1).

Considering this, the present review critically examines the mechanisms and consequences of HIV pathogenesis, with a particular emphasis on the drivers of chronic inflammation in the ART era. We explore key molecular and cellular pathways, including innate immune sensing, regulatory T-cell dysfunction, microbial translocation, and inflammasome activation, that underlie persistent immune activation despite viral suppression. Furthermore, we highlight how HIV-induced immunometabolic reprogramming, particularly in monocytes, T cells, and other immune effectors, sustains a state of low-grade inflammation and immune exhaustion. Such immunometabolic dysfunction not only impairs immune restoration but also contributes to the development and progression of both infectious and non-infectious comorbidities. Finally, we present HIV as a model for understanding the inflammatory theory of disease, offering insights into how chronic immune dysregulation drives multisystem pathology in virally suppressed individuals.

## 2. Mechanisms of Chronic Immune Activation in HIV

- Pre-ART vs. ART era changes

Although immune activation is a normal and necessary response to viral infections, this response becomes chronic, excessive, and ultimately pathogenic in the context of HIV. During the pre-ART era, persistent immune activation is primarily driven by uncontrolled HIV replication, with direct and continuous damage to the immune system. This results in massive CD4⁺ T-cell depletion, especially in the gut-associated lymphoid tissue, leading to significant immune dysfunction [20]. Here, elevated viral loads are strongly associated with increased systemic inflammation, immune cell turnover, and sustained type I interferon (IFN-I) signaling, all contributing to progressive immune exhaustion and disease advancement [21,22]. There is also a strong correlation between the degree of immune activation and HIV disease progression (independent of viral load) that underscores the crucial role of inflammation in HIV pathogenesis [19,22]. Thus, the main driver of immune activation in HIV infection during the pre-ART era is persistent HIV replication, particularly in tissue sites like lymph nodes and the gastrointestinal tract, even at very low levels. Such persistent replication together with microbial translocation and other factors leads to chronic immune activation and inflammation [23].

In contrast, many individuals exhibit persistent immune activation and inflammation during the ART era (even with long-term viral suppression), albeit through non-viral mechanisms. These include microbial translocation from damaged gut epithelia that can stimulate Toll-like receptor (TLR) pathways and monocyte activation [9,20,24], low-level HIV transcription or antigen production from latent viral reservoirs [25], and immune dysregulation, characterized by senescent and exhausted T cells [26], expanded CD14⁺CD16⁺ monocyte populations, and an altered cytokine milieu [5,9,27]. This represents a shift from direct viral cytopathic to chronic, low-grade immune dysregulation, which underlies the development of non-AIDS comorbidities such as CVD, neurocognitive impairment, and metabolic disorders in virally suppressed individuals [28,29,30].

- Cellular and Molecular Pathways

HIV infection is characterized by persistent immune activation and chronic inflammation that often persist despite ART. Unlike acute viral infections where inflammation subsides following viral clearance, HIV triggers a prolonged inflammatory response that disrupts immune homeostasis and contributes to a spectrum of non-AIDS comorbidities, particularly CVD and neurocognitive impairment [9,11]. This sustained immune activation stems from multiple molecular and cellular pathways that fuel chronic inflammation and prevent full immune restoration [20,21].

A central mechanism is HIV’s ability to evade immune clearance while persistently stimulating innate immune responses. HIV-derived RNA and reverse-transcribed DNA are recognized by pattern recognition receptors (PRRs), including TLRs and cytosolic DNA sensors of the cyclic GMP-AMP synthase (cGAS)–stimulator of interferon genes (STING) pathway [22,23]. Engagement of these receptors activates transcription factors such as nuclear factor kappa-light-chain-enhancer of activated B cells (NF-κB) and interferon regulatory refactor-3 (IRF3) resulting in the production of inflammatory cytokines like tumor necrosis factor-alpha (TNF-α), interleukin-6 (IL-6), and IFN-I [24]. While essential for antiviral defense, chronic exposure to such cytokines leads to systemic immune activation, T-cell exhaustion, and tissue injury, contributing to comorbidities (despite ART) [25].

The second major driver of chronic inflammation is gut mucosal barrier disruption. Here, HIV preferentially depletes CD4⁺ T cells in gut-associated lymphoid tissue (GALT), leading to epithelial damage and impaired immune containment. This permits microbial translocation, where bacterial products such as lipopolysaccharide (LPS), flagellin, and bacterial DNA enter systemic circulation and activate monocytes and macrophages via TLR signaling [26,27]. This results in elevated production of IL-1β, IL-18, and TNF-α, further amplifying inflammation [1,28].

Among the immune cells driving ART era inflammation, monocytes/macrophages are especially significant. Circulating CD14⁺CD16⁺ monocytes exhibit a pro-inflammatory phenotype with relatively high expression of IL-6, TNF-α, and IL-1β [29,30]. These activated monocytes contribute to vascular inflammation, endothelial dysfunction, and atherosclerotic plaque formation, playing a central role in HIV-associated CVD [31,32]. Simultaneously, plasmacytoid dendritic cells (pDCs) and regulatory T cells (Tregs) also display functional alterations, with pDCs sustaining IFN-α production [31]. Furthermore, a detrimental role for Tregs during HIV infection was suggested based on the evidence that they can suppress virus-specific immune responses [32]. Tregs often become dysfunctional or numerically depleted, which diminishes their immunoregulatory capacity [33].

**Figure 2:**
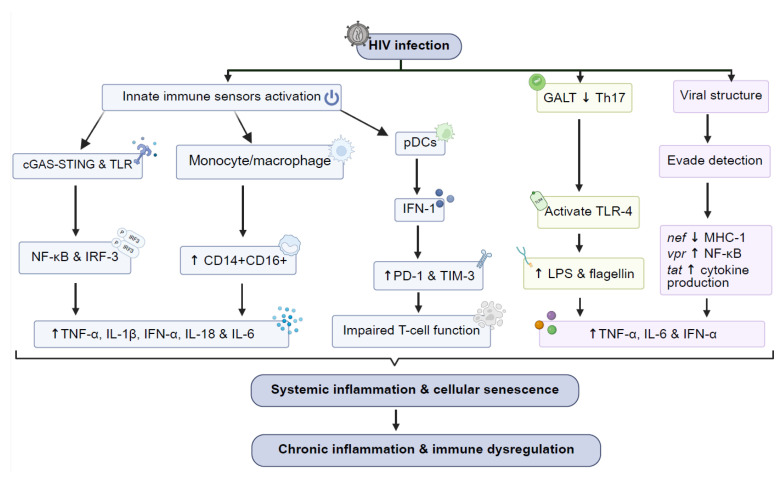
The cellular and molecular pathways driving chronic inflammation in HIV infection, featuring the activation of innate immune sensors and transcription factors (e.g., NF-κB), the upregulation of pro-inflammatory monocyte subsets (CD14⁺CD16⁺), persistent secretion of interferons, microbial translocation, and the impact of viral proteins—all contributing to systemic inflammation and immune dysregulation. Arrows in the figure represent activation, upregulation (↑), or downregulation (↓) of immune mediators and pathways involved in HIV-related immune dysregulation. Abbreviations—HIV: human immunodeficiency virus; cGAS: cyclic guanosine monophosphate/adenosine monophosphate synthase; STING: stimulator of interferon genes; TLRs: Toll-like receptors; pDCs: plasmacytoid dendritic cells; NF-κB: nuclear factor kappa B; IRF3: interferon regulatory factor 3; IFN-1: type 1 interferon; CD: cluster of differentiation; PD-1: programmed cell death protein 1; TIM-3: T-cell immunoglobulin mucin-3; TNF-α: tumor necrosis factor-alpha; IL-1β: interleukin 1-beta; IL-18: interleukin 18; IL-6: interleukin 6; IFN-α: interferon-alpha; GALT: gut-associated lymphoid tissue; Th17: T-helper 17; TLR-4: Toll-like receptor 4; LPS: lipopolysaccharide; *nef*: negative regulating factor; *vpr*: virus protein r; *tat*: transcription activator protein; and MHC I: major histocompatibility complex I. Image created using Biorender.

Importantly, chronic immune activation is not limited to the systemic circulation. It extends to tissue-specific compartments, such as the central nervous system, where ongoing glial cell activation, cytokine accumulation in cerebrospinal fluid, and monocyte trafficking contribute to neuroinflammation and the development of HIV-associated neurocognitive disorders (HANDs) [34]. This dual-axis systemic and tissue-specific immune dysregulation reflects the complex, multifactorial nature of HIV-associated inflammation in the ART era and underscores the need for integrated therapeutic strategies that go beyond viral suppression (Figure 2). In addition to microbial translocation and immune dysregulation, innate sensing of HIV-derived nucleic acids plays a vital role in perpetuating chronic inflammation. Even with suppressive ART, HIV RNA transcription continues at low levels (in latent reservoirs) and such residual viral RNA can be sensed by PRRs in immune cells. In myeloid cells and T cells, receptors such as RIG-I-like receptors and TLR7 can detect HIV single-stranded RNA, triggering downstream signaling via mitochondrial antiviral signaling protein (MAVS) and myeloid differentiation primary response 88 (MyD88), respectively, leading to the production of IFN-I and pro-inflammatory cytokines [35,36]. Such chronic low-level stimulation can maintain a persistent innate immune activation state in HIV+ persons despite viral suppression, as a result, there is a lack of pharmacologic agents that can fully block HIV RNA transcription [37].

Moreover, inflammasome activation (a significant innate immune mechanism) is another contributor to HIV-associated inflammation. Here, the caspase recruitment domain containing protein 8 (CARD8) inflammasome was recently identified as an HIV-specific sensor capable of detecting HIV protease activity in infected CD4⁺ T cells. This subsequently leads to pyroptosis, an inflammatory form of cell death characterized by IL-1β and IL-18 release [38]. This process further increases inflammation and tissue damage. Interestingly, in natural hosts of simian immunodeficiency virus (e.g., sooty mangabeys), a lack of chronic immune activation may occur due to limited inflammasome activation. This includes the relatively low expression or activity of CARD8 and NLR family pyrin domain containing 3 (NLRP3) components, contributing to their non-pathogenic course of infection [39]. These findings highlight the importance of intracellular viral sensing and inflammasome biology in sustaining inflammation, even in the absence of viremia, and provide novel insights into potential therapeutic targets for immune modulation in ART-treated HIV infection.

The IFN-I pathway is another important mechanism that enhances chronic inflammation in HIV. While IFN-α responses are crucial during early infection and particularly for viral suppression, their persistent activation during chronic HIV infection contributes to immune exhaustion, impaired T-cell function, and generalized immune dysfunction [33,34]. Plasmacytoid dendritic cells (pDCs) are the primary source of IFN-α, producing it in response to TLR7/9-mediated sensing of HIV RNA. This leads to the upregulation of inhibitory receptors such as programmed cell death 1 (PD-1) and T-cell immunoglobulin mucin-3 (TIM-3) on effector T cells, thereby limiting their ability to eliminate infected cells and exacerbating immune dysfunction [35].

In contrast, IFN-β (another key IFN-I type) is primarily produced by myeloid cells (including macrophages and monocyte-derived dendritic cells) following cytosolic sensing of HIV RNA and reverse-transcribed DNA through pathways such as RIG-I and cGAS-STING. IFN-β plays a broader and earlier role in the innate antiviral response. Of note, while IFN-α production by pDCs is prominent during acute infection, their functional contribution during ART-mediated viral suppression remains less clear. pDCs are often numerically depleted or functionally impaired in people living with HIV on long-term ART. However, residual or aberrant pDC activation that is potentially triggered by low-level HIV transcription or microbial products may still sustain chronic low-grade IFN signaling even when plasma viremia is undetectable [40,41]. Thus, instead of supporting durable antiviral immunity, chronic IFN-I signaling (both IFN-α and IFN-β) can drive systemic inflammation, disrupt immune homeostasis, and promote immune senescence, further contributing to HIV-associated comorbidities [33,36] (Figure 2).

The structural composition of HIV plays a fundamental role in its ability to escape immune detection, establish persistent infection, and sustain chronic immune activation. HIV is an enveloped retrovirus with key structural components that interact directly with the host immune system and influence disease progression. The envelope glycoproteins gp120 and gp41 are crucial for HIV entry into host cells. Here, gp120 can bind to the CD4 receptor on T cells and co-receptors, i.e., C-C chemokine receptor type 5 (CCR5) or C-X-C chemokine receptor type 4 (CXCR4), to facilitate viral entry. However, gp120 is highly glycosylated, creating a “glycan shield” that hides the virus from antibody recognition [42]. Additionally, rapid mutations in gp120 help HIV evade neutralizing antibodies and make it difficult for the immune system to mount an effective long-term response [42,43]. Although the p24 capsid protein (which encases viral RNA) is also highly immunogenic and serves as a major target for immune recognition, HIV has evolved mechanisms to modulate capsid detection. The viral capsid can influence the host’s innate immune sensing pathways, particularly by interacting with cytosolic sensors (e.g., cyclic guanosine monophosphate/adenosine monophosphate synthase–stimulator of interferon genes pathway) which usually detect viral DNA. Moreover, some HIV strains mutate their capsid structure to evade detection, thereby delay the immune response, and hence allow for viral persistence [44].

HIV also carries reverse transcriptase that converts viral RNA into DNA and is subsequently integrated into the host genome by integrase. Such integration establishes a lifelong infection and allows HIV to persist despite immune surveillance and clearance mechanisms. The persistent presence of viral DNA within immune cells stems from both reverse-transcribed HIV cDNA and integrated proviral DNA, each contributing to chronic immune activation even in the context of suppressive ART [45]. Reverse-transcribed HIV cDNA (generated early during the infection cycle) can transiently accumulate in the cytosol and trigger innate immune sensors such as cGAS, which can activate the STING pathway and promote the release of IFN-I and pro-inflammatory cytokines [46]. However, integrated proviral DNA is stably inserted within the host genome and may stay transcriptionally active especially in non-resting CD4⁺ T cells. Even though most integrated proviruses are defective, a subset can still produce low-level HIV RNA and proteins that can stimulate immune cells via PRRs, including TLRs, RIG-I, and MDA5. Such intermittent viral outputs can act as chronic immunostimulatory signals to reinforce immune activation and inflammation in virally suppressed individuals [47,48]. Such integration establishes a lifelong infection and allows the virus to persist despite immune clearance efforts. The persistent presence of viral DNA within immune cells can also fuel chronic immune activation even in the presence of ART [48] (Figure 2).

Viral proteins such as Nef, Vpr, and Tat also actively contribute to inflammation. For example, the Nef protein of HIV-1 downregulates major histocompatibility complex class I expression on infected cells, which allows the virus to evade the immune system and to persist. This process impairs immune surveillance and can lead to prolonged immune activation [49,50]. The Vpr protein can trigger apoptosis by inducing DNA damage, which in turn activates inflammatory pathways like NF-κB. This can lead to cellular senescence and a state of permanent cell cycle arrest and thereby promote the detrimental effects of HIV infection on the host’s immune system [51]. Tat can also enhance pro-inflammatory cytokine production and disrupt the blood–brain barrier, leading to neuroinflammation and the development of HANDs [52]. Such viral proteins further exacerbate immune dysfunction, creating a cycle of immune activation, inflammation, and tissue damage that persists even when HIV replication is effectively suppressed by ART [53]. This chronic inflammatory state not only promotes HIV-related complications but also adds to the broader inflammatory theory of disease, linking HIV infection to conditions such as CVD, metabolic disorders, and neurodegenerative diseases.

HIV infection therefore serves as a powerful model to highlight the inflammatory theory of disease as its molecular pathways directly sustain a chronic inflammatory state [7,54,55]. Persistent immune activation during HIV indeed reflects inflammatory processes observed in conditions such as atherosclerosis, cancer, and autoimmune diseases and reinforces the concept that inflammation is not a consequence but rather a driver of disease progression [12,54,56]. The interplay between gut barrier dysfunction, innate immune activation, persistent IFN signaling, and viral protein-induced inflammation demonstrates how a single infection can lead to widespread immune dysregulation, enhancing both immune exhaustion and long-term tissue damage [12,54,57,58]. A greater understanding of these molecular pathways is important for developing targeted interventions to decrease chronic inflammation in people living with HIV (PLWH), not only to improve immune recovery but also to mitigate the wider pathological consequences of persistent immune activation.

## 3. Immune Cell Dysfunction and Senescence in HIV

- Imbalance Between Pro- and Anti-Inflammatory Responses

HIV infection is marked by persistent immune activation and a disrupted balance between pro- and anti-inflammatory responses. This imbalance exacerbates immune dysfunction and contributes to the development of non-AIDS comorbidities, including cardiovascular and neurocognitive diseases. Regulatory T cells (Tregs), which are central to maintaining immune homeostasis by suppressing excessive inflammation, become both functionally impaired and numerically depleted in PLWH [5,50]. Despite their crucial role, Tregs fail to effectively control the heightened activation of effector T cells and monocytes, resulting in sustained production of pro-inflammatory cytokines such as TNF-α, IL-6, IL-1β, and IFN-α [51].

Several mechanisms underlie Treg dysfunction in HIV. Although Tregs are less permissive to HIV replication than activated CD4⁺ T cells, they express both CD4 and CCR5, rendering them susceptible to direct infection and apoptosis, particularly during early infection [32,59]. This leads to a significant depletion of Tregs, especially within the GALT [60]. In addition, the pro-inflammatory milieu characterized by elevated IL-6, IL-1β, and TNF-α destabilizes the Treg phenotype and promotes their conversion into pro-inflammatory IL-17⁺ “ex-Tregs” with diminished suppressive capacity [61]. Treg homeostasis also depends on low-dose IL-2 signaling, which becomes dysregulated in HIV due to a cytokine imbalance and competition with activated effector T cells, leading to Treg exhaustion [59,62]. Furthermore, microbial translocation following gut barrier disruption introduces bacterial products such as LPS into circulation, activating TLR pathways on antigen-presenting cells and Tregs alike, further reducing their regulatory function [20,62]. Moreover, Tregs may be redistributed from circulation to inflamed tissues such as the central nervous system and vascular endothelium, contributing to peripheral depletion and local immune dysregulation [63,64,65].

Collectively, the loss and dysfunction of Tregs in HIV infection compromise immune regulation, allowing for unchecked activation of effector T cells and monocytes. This sustains a cycle of chronic inflammation even in individuals on effective ART and plays a key role in driving HIV-associated comorbidities [11,66].

In parallel, overactivation of inflammatory pathways (e.g., NF-κB and IFN-I signaling cascades) can exacerbate tissue damage and immune exhaustion [67,68]. Effector immune cells (particularly CD8^+^ T-cells and monocytes) remain in a hyperactivated state but gradually lose their functional capacity due to persistent antigen exposure [69]. This phenomenon is known as immune exhaustion and mirrors inflammatory processes observed in other chronic diseases such as cancer and autoimmune disorders. Unlike the phenomenon observed in tumors where excessive Treg activity suppresses anti-tumor immunity [70], a similar but opposite effect occurs in HIV where insufficient Treg-mediated regulation fails to resolve systemic inflammation, leading to widespread immune dysfunction [19]. In addition, anti-inflammatory cytokines such as IL-10 and transforming growth factor-beta (TGF-β) are insufficient to counteract this overwhelming inflammatory response [70]. The defective anti-inflammatory mechanisms in HIV not only prolong inflammation but also contribute to immune exhaustion where hyperactivated T cells progressively lose their functional capacity, further exacerbating immune dysregulation [11,12].

This imbalance between pro- and anti-inflammatory responses in HIV aligns with the broader inflammatory theory of disease as it mirrors patterns observed in non-communicable inflammatory conditions. The uncontrolled inflammation in HIV-infected individuals accelerates aging-related diseases, including CVD, insulin resistance, and neuroinflammation, resembling processes observed in autoimmune diseases and chronic metabolic syndromes [54,71]. The inability of Tregs to restore immune balance in HIV-infected individuals further underscores the interconnected nature of chronic inflammatory diseases, linking infectious and non-infectious conditions in a more unified framework [72]. Understanding this immunological imbalance in HIV provides a unique opportunity to explore therapeutic interventions aimed at restoring immune homeostasis. Strategies targeting Treg function, metabolic pathways, and inflammatory cytokine modulation may not only improve immune regulation in PLWH but also offer insights into broader approaches for managing chronic inflammatory diseases. Thus, by integrating HIV-induced immune dysregulation into the inflammatory theory of disease, we gain a deeper understanding of how chronic inflammation acts as a central mechanism underlying a diverse array of pathologic complications.

- HIV-Induced Senescence and the Senescence-Associated Secretory Phenotype

Cellular senescence is a state of permanent cell cycle arrest triggered by various stressors such as chronic immune activation, oxidative stress, and DNA damage [57,58]. Although initially protective (preventing the proliferation of damaged cells), senescent cells often acquire a pro-inflammatory phenotype known as the senescence-associated secretory phenotype (SASP). This phenotype is characterized by the secretion of pro-inflammatory cytokines (e.g., IL-6, IL-1β, TNF-α), chemokines, and growth factors that promote tissue remodeling, chronic inflammation, and age-related pathology [59,60].

In the context of HIV infection, persistent immune activation and systemic inflammation accelerate cellular senescence particularly in CD4⁺ and CD8⁺ T cells, monocytes, and endothelial cells, contributing to premature immune aging or inflammaging [61]. This process, often termed “HIV-induced senescence,” results from a combination of chronic immune stress and the direct actions of viral proteins such as Tat and Nef, which impair DNA repair mechanisms, mitochondrial function, and cell cycle regulation [73,74,75]. Such insults lead to hallmark features of senescence, including DNA damage, telomere abrasion, and epigenetic reprogramming [76].

Senescent T cells exhibit reduced proliferative capacity, upregulation of inhibitory receptors such as PD-1 and TIM-3, and a diminished ability to respond to new infections or vaccines [62]. Although such dysfunction mirrors the immune aging found in older adults, it occurs at an accelerated rate in HIV-infected individuals, even in those on long-term ART [77]. Beyond T cells, senescent monocytes and macrophages contribute to systemic inflammation by producing elevated levels of inflammatory cytokines, which are linked to vascular inflammation and an increased risk of CVD, atherosclerosis, and metabolic complications in PLWH [64].

Senescence also affects non-immune cells, including stromal and endothelial cells, contributing to tissue fibrosis, neuroinflammation, and end-organ damage. For example, senescent endothelial cells lining the vasculature demonstrate increased permeability and impaired function, promoting hypertension and atherosclerosis, both of which are more prevalent in HIV-infected individuals than in the general population [65,66]. In the central nervous system, senescent astrocytes and microglia contribute to HIV-associated neuroinflammation, which is implicated in cognitive decline and neurodegenerative conditions such as HANDs and Alzheimer’s disease [67].

The interaction between HIV-induced senescence and age-related diseases further supports the inflammatory theory of disease, which posits that chronic, low-grade inflammation is a central mechanism driving both immune dysfunction and systemic comorbidities [19]. This premature aging phenotype underscores the need for targeted therapeutic strategies that aim to reduce senescence, control SASP, and alleviate chronic inflammation. Emerging approaches including senolytic agents that selectively eliminate senescent cells, anti-inflammatory therapies, and metabolic modulators show promise in improving long-term health outcomes not only for PLWH but also for aging populations at large. By mitigating senescence-associated inflammation, these interventions may help prevent the progression of a wide range of chronic inflammatory diseases [68,69].

## 4. Immunometabolic Reprogramming in HIV Infection

Immunometabolism refers to the dynamic interplay between cellular metabolism and immune function, shaping how immune cells respond to infections, inflammation, and disease progression [5,78]. With HIV infection, metabolic dysregulation is closely linked to persistent immune activation, chronic inflammation, and immune dysfunction. These metabolic alterations contribute to disease progression and comorbidities such as CVD, metabolic disorders, and neuroinflammation [11,79]. Understanding such shifts provides crucial insights into how HIV-induced immune activation supports the inflammatory theory of disease.

I. Metabolic Reprogramming of Immune Cells in HIV

HIV infection profoundly alters the metabolic landscape of immune cells, particularly T cells, monocytes, and macrophages. This metabolic reprogramming fuels persistent immune activation and dysfunction and adds to the systemic inflammatory state observed in PLWH. For example, T cells rely on aerobic glycolysis (the Warburg effect) to meet the energy demands of rapid proliferation and effector functioning. However, this metabolic shift (crucial for mounting an immune response) becomes dysregulated with HIV infection due to continuous immune activation [80]. Over time, this persistent stimulation leads to metabolic exhaustion, with CD4^+^ and CD8^+^ T cells now characterized by impaired mitochondrial function, an overproduction of reactive oxygen species, and diminished energy reserves [79,80,81]. As a result, such exhausted T cells lose their ability to respond effectively. This leads to progressive immune senescence and apoptosis that can accelerate disease progression [81,82]. Another important aspect of T-cell dysfunction is the impairment of Tregs which typically help suppress excessive immune activation. Unlike effector T cells, Tregs primarily depend on oxidative phosphorylation for energy [82]. However, HIV-induced metabolic disruptions compromise Treg function and diminish their ability to regulate inflammation. This imbalance further perpetuates the cycle of immune activation and chronic inflammation and creates a self-sustaining loop that underlies HIV pathogenesis.

T-cell exhaustion was initially described in chronic viral infections like lymphocytic choriomeningitis virus [83]. It is a hallmark of HIV infection, where it contributes to progressive immune dysfunction. With chronic HIV infection, persistently high levels of viral replication drive CD8^+^ T cells into an exhausted state and impair their ability to control the virus. This exhaustion is exacerbated by chronic immune activation and disrupted T-cell homeostasis [84]. A key marker of T-cell exhaustion in HIV is the upregulation of PD-1 on virus-specific T cells, which correlates with disease progression, impaired T-cell function, higher viral loads, and declining CD4^+^ T-cell counts [85]. Unlike HIV-specific T cells, cytomegalovirus-specific T cells in the same individuals do not exhibit this upregulation, thereby highlighting the HIV-driven nature of exhaustion. As PD-1 expression gradually declines with ART, this indicates partial immune recovery [84,85,86]. Other inhibitory receptors, including lymphocyte activation gene 3, TIM-3, T-cell immunoglobulin and ITIM domain, 2B4/CD244, and CD160, also contribute to more severe stages of exhaustion. The co-expression of PD-1 with T-cell immunoglobulin and ITIM domain or lymphocyte activation gene 3 correlates with disease progression, while multiple inhibitory markers together predict lowered T-cell functionality [85,86]. These findings underscore the importance of targeting immune checkpoints to restore T-cell function and enhance immune responses in PLWH.

Monocytes and macrophages are essential components of the innate immune system and undergo profound metabolic shifts in response to HIV infection. Here, such cells are reprogrammed to rely more on glycolysis (versus mitochondrial respiration for energy production), a metabolic adaptation that stimulates a pro-inflammatory phenotype [81,87]. This shift enhances their ability to secrete large amounts of pro-inflammatory cytokines, including IL-1β, TNF-α, and IL-6 that promote sustained systemic inflammation [88]. The metabolic dysfunction in monocytes and macrophages extends beyond cytokine production. Here, persistent activation of such cells contributes to the heightened risk of CVD and metabolic disorders commonly observed in PLWH [79,89]. Moreover, chronic inflammation driven by monocyte activation is robustly linked to the development of atherosclerosis, insulin resistance, and other metabolic complications [90]. Mitochondrial dysfunction in macrophages can also exacerbate oxidative stress and tissue damage, impairing their ability to effectively regulate immune responses [91]. This metabolic maladaptation not only fuels ongoing inflammation but also compromises the overall immune environment, further amplifying HIV-associated comorbidities [92]. Collectively, the metabolic reprogramming of T cells, monocytes, and macrophages in HIV infection can establish a state of persistent immune activation that promotes chronic inflammation and immune dysfunction. Such metabolic alterations provide key mechanistic insights into how HIV drives disease progression and underscores the critical role of immunometabolism in understanding the inflammatory theory of disease [5,11].

II. Glycolysis, Chronic Inflammation, and the Inflammatory Theory of Disease

The metabolic reprogramming of immune cells that fuels sustained immune activation and tissue damage is central to this inflammatory paradigm. Here, glycolysis is a crucial mediator of this metabolic shift [93] as immune cells increasingly favor it over oxidative phosphorylation in the context of chronic inflammatory diseases, a phenomenon akin to the Warburg effect observed in cancer [88,94]. This glycolytic preference sustains a pro-inflammatory state and reinforces the chronic inflammation found in many metabolic disorders and CVD [88].

Metabolic diseases such as obesity, diabetes, and metabolic syndrome exemplify the inflammatory theory of disease, where chronic low-grade inflammation (often termed metaflammation) enables disease progression. The metabolic reprogramming of adipose tissue macrophages is a major contributor to persistent inflammation [95]. As the adipose tissues expand, adipose tissue macrophages transition from an anti-inflammatory (macrophage type 2; M2) phenotype to a pro-inflammatory (macrophage type 1-M1) state, characterized by heightened glycolysis [96]. This metabolic shift supports the production of inflammatory cytokines (e.g., TNF-α, IL-6, IL-1β) which promote systemic inflammation, insulin resistance, and endothelial dysfunction [96].

At the molecular level, hypoxia-inducible factor-1α (HIF-1α) (a master regulator of glycolysis) is upregulated in inflamed tissues. When tissues become inflamed the relatively lower oxygen levels trigger an increase in HIF-1α expression as it is a key regulator of the cellular response to hypoxia. HIF-1α subsequently elevates the expression and production of glycolytic enzymes, leading to enhanced glycolysis within the inflamed area. This in turn enhances the inflammatory response by facilitating the production of inflammatory cytokines and essentially amplifying the inflammatory cascade at the molecular level [97]. Furthermore, metabolic regulators such as 5′ adenosine monophosphate-activated protein kinase (AMPK) and mammalian target of rapamycin (mTOR) can influence the interplay between glycolysis and immune activation, linking intracellular energy balance to inflammatory signaling [98]. The persistent activation of such pathways contributes to the systemic inflammatory characteristics of metabolic diseases, aligning with the broader inflammatory theory that chronic immune activation underlies disease progression.

Atherosclerosis is a prime example of an inflammation-driven disease and further demonstrates the role of increased glycolysis in immune activation and disease progression. Chronic vascular inflammation is initiated and sustained by the metabolic reprogramming of monocytes and macrophages. Upon recruitment to the vascular endothelium, monocytes differentiate into macrophages, engulf oxidized low-density lipoprotein (ox-LDL), and form foam cells [99,100]. Such cells rely heavily on glycolysis to sustain their inflammatory activity and produce relatively high levels of IL-1β, TNF-α, and monocyte chemoattractant protein-1, which can promote plaque formation and instability [99,100]. An important component of this inflammatory cascade is the NLRP3 inflammasome, an innate immune sensor that is closely tied to glycolytic metabolism. Elevated glycolysis generates ATP and key metabolic intermediates such as succinate that can both stabilize HIF-1α and also enhance IL-1β production [101,102]. The inflammatory feedback loop in atherosclerosis refers to a cycle where the initial damage to the endothelium triggers an inflammatory response, which then further damages the endothelium. This leads to smooth muscle cell proliferation, plaque formation, and ultimately plaque rupture, which are key characteristics of advanced atherosclerosis [103]. The persistence of this inflammatory state (even in the absence of an acute infection and/or injury) underscores the inflammatory theory of disease by linking metabolic dysregulation to chronic inflammatory pathology.

The chronic inflammatory states observed both in metabolic disorders and atherosclerosis exemplify how immune metabolism (particularly enhanced glycolysis) contributes to the inflammatory theory of disease. Instead of resolving inflammation, such metabolic reprogramming in immune cells sustains a pathological inflammatory response and hence perpetuates disease progression. Such insights allow for the pursuance of novel therapeutic avenues where metabolic interventions—such as AMPK activators (metformin), mTOR inhibitors (rapamycin), and glycolysis inhibitors (2-deoxyglucose)—may help disrupt chronic inflammatory cycles and mitigate disease burden [104]. Targeting glycolytic metabolism not only offers potential treatments for metabolic and CVD, but also strengthens the broader concept that inflammation is a fundamental driver of chronic disease pathology.

III. The Role of Lipid Metabolism in HIV-Driven Inflammation

Lipid metabolism plays a crucial role in the inflammatory processes associated with HIV infection, contributing to immune activation, viral persistence, and an increased risk of metabolic disorders. One of the most significant alterations occurs in cholesterol metabolism, where HIV-infected immune cells exhibit abnormal lipid accumulation. This is particularly evident in macrophages which engulf excess lipids and transform into foam cells—a well-described hallmark feature of atherosclerosis. The formation of foam cells promotes vascular inflammation and plaque development, significantly increasing the risk of CVD in PLWH [104,105]. HIV also affects fatty acid metabolism to further enhance chronic inflammation. As fatty acids serve as key bioactive molecules in immune signaling, their dysregulation creates an inflammatory microenvironment that supports viral persistence and immune activation. Here, infected immune cells such as macrophages and T cells exhibit altered fatty acid oxidation, leading to a shift toward a pro-inflammatory phenotype. This metabolic state favors the continuous activation of immune pathways and reinforces systemic inflammation and the impairment of immune resolution mechanisms [11,92].

HIV infection is also associated with elevated levels of ceramides and sphingolipids, lipid molecules that play significant roles in cell signaling, inflammation, and metabolic regulation [106]. For example, increased ceramide accumulation has been linked to immune activation, insulin resistance, and metabolic syndrome, contributing to the increasing prevalence of diabetes and other metabolic disorders in PLWH [105,107]. Such lipid-driven inflammatory pathways further integrate HIV-associated inflammation with broader metabolic dysfunction and highlight the intersection between lipid metabolism and chronic disease progression in HIV infection [108,109]. HIV infection and the subsequent provision of ART are often associated with perturbations in lipid profiles. Here, persistent inflammation continues to drive modifications in lipid composition and function (despite viral replication suppression by ART), thereby exacerbating CVD risk [107]. Increased levels of several pro-inflammatory lipid species, including ox-LDL and high-density lipoprotein (HDL), have been detected in HIV-infected individuals and are associated with markers of immune activation [108]. This bidirectional relationship, where inflammation increases lipid levels and promotes their modification, while such altered lipid species further sustain inflammatory processes, requires further investigation.

Addressing lipid metabolism disturbances is therefore important when aiming to lower CVD risk in PLWH. Treatment with statins and lifestyle modifications (such as improvements in diet and exercise) have shown potential in mitigating such risks [108]. However, well-designed clinical trials that consider the complex interactions between lipids and inflammation in HIV infection are necessary to develop targeted therapeutic strategies.

## 5. Gut Microbiome, Immune Dysregulation, and Metabolite Changes

The gastrointestinal tract plays a central role in immune regulation, and its integrity is severely compromised during HIV infection. One of the earliest and most profound effects of HIV is damage to gut-associated lymphoid tissue, which serves as a key site for immune surveillance and homeostasis. The depletion of intestinal CD4^+^ T cells (particularly Th17 cells) weakens the gut barrier and leads to increased permeability and the translocation of microbial products into circulation. Such microbial translocation is a major driver of systemic inflammation in PLWH [110,111]. One of the primary consequences of microbial translocation is the increased presence of bacterial endotoxins such as LPS in the bloodstream. LPS strongly activates TLR4 on immune cells and can trigger a pro-inflammatory cascade that perpetuates chronic immune activation [112]. This mechanism contributes not only to HIV-associated inflammation but also applies to the broader CVD context as well as metabolic disturbances such as insulin resistance.

**Figure 3:**
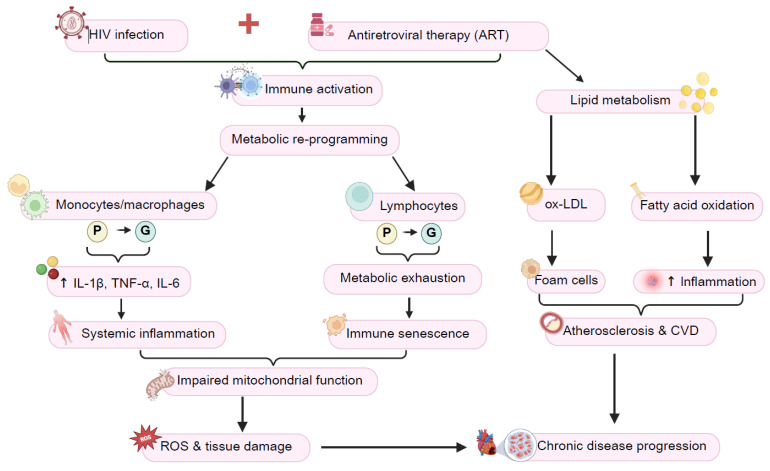
Metabolic reprogramming of T cells, monocytes, and macrophages during HIV infection drives persistent immune activation that can lead to sustained chronic inflammation, immune dysfunction, and disease progression. Abbreviations—HIV: human immunodeficiency virus; IL-1β: interleukin-1 beta; TNF-α: tumor necrosis factor alpha; IL-6: interleukin-6; ROS: reactive oxygen species; ox-LDL: oxidized low-density lipoprotein; CVD: cardiovascular disease; 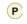: oxidative phosphorylation; and 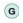: glycolysis. Image created using Biorender.

HIV infection also induces significant changes in the gut microbiome composition, leading to a state of dysbiosis, an imbalance in microbial communities [113]. Dysbiosis disrupts the host’s metabolism and contributes to abnormal glucose and lipid metabolism. For example, certain bacterial species that produce short-chain fatty acids (SCFAs) (possessing anti-inflammatory properties) are attenuated in PLWH, while pathogenic and inflammatory bacterial species become more dominant [114]. In support, Sereti et al. [115] examined microbiota composition and SCFA dynamics in serum and stool of PLWH sampled prior to the onset of comorbidities or death. Here, systemic SCFAs were significantly depleted in PWLH and were associated with prognostic inflammatory markers and decreases in gut microbial SCFA production pathways. Such alterations further exacerbate immune dysfunction, metabolic stress, and systemic inflammation, creating a vicious cycle that mirrors metabolic disturbances observed in other inflammatory diseases such as obesity, type 2 diabetes, and autoimmune disorders (Figure 3) [115].

Together, HIV infection therefore provides a unique model of the inflammatory theory of disease as it links gut dysbiosis, microbial translocation, and metabolic dysregulation. The gut’s role as both an immune and metabolic organ highlights the complex interplay between microbial health, systemic inflammation, and chronic disease progression. Understanding and targeting these interactions may offer unique therapeutic strategies to mitigate inflammation and improve long-term health outcomes in PLWH and for other chronic cardio-metabolic diseases in non-infected individuals.

## 6. Chronic Inflammation and Immune Activation in HIV-Associated Comorbidities: Focus on CVDs and HANDs

Cardiovascular diseases and HANDs are among the most clinically relevant outcomes of chronic immune activation and serve as compelling examples of how immune dysregulation and metabolic disturbances converge to drive serious comorbidities, even in virally suppressed individuals.

- Cardiovascular Diseases (CVDs) during HIV Infection

Our own research work and those of others have shown that CVD is a leading comorbidity in HIV and that this occurs not merely due to classical risk factors but largely because of immune cell dysfunction and chronic vascular inflammation. For example, HIV infection can activate monocytes/macrophages and T cells, disrupting endothelial integrity and promoting fibrosis and atherogenesis. In support, our laboratory previously demonstrated that inflammatory monocyte subsets (particularly CD14⁺CD16⁺) play a crucial role in this process as they express relatively high levels of IL-6, TNF-α, monocyte chemoattractant protein-1 (MCP-1), and tissue factor—directly linking them to coagulation and endothelial damage [5,116]. Such innate immune abnormalities are in our opinion central to understanding why PLWH remain at a relatively high CVD risk despite viral suppression.

Equally important are the immunometabolic changes we and others have described in HIV. Here, dysfunctional mitochondria and altered glucose and lipid handling in immune cells drive them toward a pro-inflammatory phenotype, perpetuating vascular injury and metabolic stress [89,117]. Moreover, we previously reported elevated levels of Glycoprotein-A repetition predominant (GARP)-expressing CD4⁺ T cells and signs of activation-induced coagulopathy, demonstrating a broader dysregulation of the immune–endothelial interface in ART-treated patients [79]. Taken together, these findings strongly support the concept that HIV-associated CVD is an immunologically mediated disease that is shaped by persistent inflammation and immune–metabolic dysfunction rather than ART exposure [118,119,120].

- HIV-Associated Neurocognitive Disorders (HANDs)

HANDs encompass a continuum of cognitive impairments ranging from asymptomatic neurocognitive impairment to HIV-associated dementia. Despite effective ART and sustained viral suppression, HANDs remain prevalent in PLWH, indicating that factors beyond active viral replication contribute to its pathogenesis. A central mechanism underlying HANDs is chronic neuroinflammation, which is driven by systemic immune activation, microbial translocation, and direct involvement of the central nervous system. This inflammatory milieu leads to activation of astrocytes and microglia (resident immune cells of the brain), which secrete pro-inflammatory cytokines and reactive oxygen species. Such mediators contribute to neuronal damage, synaptic dysfunction, and cognitive decline [121]. Low-level viral replication, residual IFN-I signaling, and chronic immune stimulation persist even during the ART era. These are reflected in elevated levels of neuroimmune activation markers, including neopterin, as well as soluble CD14 and soluble CD163, which are associated with ongoing neuroinflammatory cascades and HAND progression [122]. Emerging evidence indicates that senescent glial cells may further exacerbate HAND pathology. Both astrocytes and microglia can acquire an SASP that is characterized by the release of pro-inflammatory cytokines, chemokines, and proteases, thereby contributing to sustained inflammation and neuronal toxicity [122,123].

While the direct contribution of viral proteins such as HIV-1 Tat to neurotoxicity is still under investigation, immune-mediated mechanisms are widely recognized as the primary drivers of chronic HANDs. Moreover, neuropathological studies increasingly reveal Alzheimer’s disease-like features in the brains of PLWH, including amyloid deposition, tau phosphorylation, and neurodegeneration, suggesting overlapping mechanisms between HANDs and age-related neurocognitive disorders [124]. These findings support the view that HANDs are a consequence of chronic immune activation, neuroinflammation, and cellular senescence, rather than ongoing viral replication alone. Addressing such pathologic mechanisms will be crucial to improving cognitive outcomes in PLWH in the ART era.

From a clinical and mechanistic perspective, both CVDs and HANDs demonstrate how persistent inflammation, immune senescence, and metabolic dysfunction remain central to disease development in ART-suppressed PLWH. These comorbidities align with the inflammatory theory of disease and in our opinion reinforce the value of HIV as a model to study the intersection of immune dysfunction and age-related pathology. We therefore propose that by targeting inflammatory and immunometabolic pathways, e.g., by using senolytics, anti-inflammatory agents, and/or metabolic interventions, long-term health outcomes can be improved not only in PLWH but also in those with other complications that manifest as non-HIV chronic inflammatory diseases.

## 7. Potential Therapeutic Targets to Address HIV-Related Immunometabolism

Considering the robust links between metabolic dysfunction and inflammation in HIV, targeting immunometabolism pathways offers a promising strategy to mitigate chronic immune activation and associated comorbidities. One approach involves metformin, a widely used diabetes drug that can also enhance mitochondrial function and lower inflammation via AMPK activation. For example, some found that metformin could enhance the immune system’s ability to recognize HIV-infected cells [125]. Such research indicated that metformin treatment increased the expression of bone marrow stromal antigen 2 on productively infected T cells, thereby enhancing their (antibody-mediated) recognition. This finding suggests that metformin may aid in targeting and eliminating latent HIV reservoirs [125]. Metformin may thus help to restore the immune balance in PLWH by improving intracellular energy production and by limiting oxidative stress [92,125].

Another therapeutic strategy would be to employ mTOR inhibitors like rapamycin, which modulates T-cell metabolism and thereby prevents excessive activation and immune exhaustion. Here, such inhibitors may help to lower chronic inflammation and improve immune resilience by regulating metabolic pathways that control immune function [126,127]. Although statins are commonly prescribed for lowering cholesterol levels, they have shown benefits in terms of improving lipid metabolism and decreasing monocyte activation, thereby lowering cardiovascular risk in PLWH [128,129]. Their anti-inflammatory effects make them a promising adjunct therapy for addressing metabolic and cardiovascular complications associated with HIV [130,131]. In support, the REPRIEVE (Randomized Trial to Prevent Vascular Events in HIV) evaluated the efficacy of pitavastatin in lowering cardiovascular events among PLWH. This large-scale study enrolled 7769 HIV-positive participants with relatively low-to-moderate cardiovascular risk. These findings demonstrated that pitavastatin significantly decreased the risk (by 35%) of major adverse cardiovascular events compared to placebo over a median follow-up of 5.1 years. Importantly, pitavastatin was chosen due to its minimal interactions with ART, highlighting its suitability for this context [132].

Finally, restoring gut microbiota balance is another area of intervention to consider as pre- and probiotics have been explored for their ability to improve gut health, attenuate microbial translocation, and thereby lower systemic inflammation. In agreement, a randomized controlled trial investigating the effects of a probiotic supplement in ART-treated PLWH found that such an intervention could partially restore gut barrier integrity and lowered microbial translocation, a key driver of HIV-related systemic inflammation [133]. They also investigated whether prebiotics and symbiotics (combination of prebiotics and probiotics) could modulate gut microbiota and decrease inflammation in PLWH. Here, the preliminary results suggest beneficial effects on gut microbial balance together with decreased immune activation [134]. Thus, such therapeutic interventions offer a potential avenue for mitigating the effects of HIV-induced gut dysbiosis and inflammation by enhancing gut integrity and modulating immune responses [135]. Of note, while such research work shows promising findings, the results have been variable and the long-term clinical benefits of gut microbiota-targeted therapies in HIV remain an area of active investigation. Further research is therefore required to determine optimal probiotic strains, dosing strategies, and their sustained impact on systemic inflammation and non-AIDS comorbidities.

## 8. Conclusions

HIV infection demonstrates the intricate relationship between chronic immune activation, metabolic dysfunction, and systemic inflammation, and thus reinforces the broader inflammatory theory of disease. Here, persistent inflammation continues to drive immune dysregulation (despite effective ART) and contributes to an increased burden of cardiovascular, metabolic, and neurodegenerative diseases. The parallels between HIV-driven inflammation and other chronic inflammatory conditions underscore the need for targeted immunometabolism interventions. By dissecting the mechanisms underlying HIV-associated inflammation, we not only enhance our ability to improve health outcomes for PLWH but also gain crucial insights into therapeutic strategies that may be applicable across a spectrum of inflammation-mediated chronic diseases. Advancing our understanding of such shared pathways should help pave the way for novel treatments that transcend infectious diseases and that begin to address the root causes of chronic inflammation in numerous pathological states, as discussed in this review article.
